# From Structure to Function: How Prebiotic Diversity Shapes Gut Integrity and Immune Balance

**DOI:** 10.3390/nu16244286

**Published:** 2024-12-12

**Authors:** Lucas de Freitas Pedrosa, Paul de Vos, João Paulo Fabi

**Affiliations:** 1 Department of Food Science and Experimental Nutrition, School of Pharmaceutical Sciences, University of São Paulo, São Paulo 05508-000, SP, Brazil; lfpedrosa@usp.br; 2 Department of Pathology and Medical Biology, University Medical Center Groningen, University of Groningen, 9713 GZ Groningen, The Netherlands; p.de.vos@umcg.nl; 3 Food and Nutrition Research Center (NAPAN), University of São Paulo, São Paulo 05508-000, SP, Brazil; 4 Food Research Center (FoRC), CEPID-FAPESP (Research, Innovation, and Dissemination Centers), São Paulo 05508-080, SP, Brazil; 5 Food Research Center (FoRC), CEPIX-USP, University of São Paulo, São Paulo 05508-000, SP, Brazil

**Keywords:** microbiota, prebiotics, inflammation, polysaccharides, dysbiosis

## Abstract

The microbiota stability, diversity, and composition are pillars for an efficient and beneficial symbiotic relationship between its host and itself. Microbial dysbiosis, a condition where a homeostatic bacterial community is disturbed by acute or chronic events, is a predisposition for many diseases, including local and systemic inflammation that leads to metabolic syndrome, diabetes, and some types of cancers. Classical dysbiosis occurs in the large intestine. During this period, pathogenic strains can multiply, taking advantage of the compromised environment. This overgrowth triggers an exaggerated inflammatory response from the human immune system due to the weakened integrity of the intestinal barrier. Such inflammation can also directly influence higher polyp formation and/or tumorigenesis. Prebiotics can be instrumental in preventing or correcting dysbiosis. Prebiotics are molecules capable of serving as substrates for fermentation processes by gut microorganisms. This can promote returning the intestinal environment to homeostasis. Effective prebiotics are generally specific oligo- and polysaccharides, such as FOS or inulin. However, the direct effects of prebiotics on intestinal epithelial and immune cells must also be taken into consideration. This interaction happens with diverse prebiotic nondigestible carbohydrates, and they can inhibit or decrease the inflammatory response. The present work aims to elucidate and describe the different types of prebiotics, their influence, and their functionalities for health, primarily focusing on their ability to reduce and control inflammation in the intestinal epithelial barrier, gut, and systemic environments.

## 1. Introduction

The correlations among dietary components, gut microbiota modulation, and the resulting health-related effects are a constant topic of interest throughout the scientific community. Prebiotics are one type of dietary component that is notably explored. Prebiotics might stimulate the activity or growth of certain bacterial strains, specifically fermented by local microbiota, which can establish or strengthen a symbiotic relationship between microbiota and the host [1].

Prebiotics support intestinal health by maintaining, repairing, and straightening the gut barrier. This barrier is a complex and multifaceted structure comprised of a protective mucus layer, a tightly regulated epithelial cell layer, and specialized proteins known as tight junctions, which act as gatekeepers for the gut lining. Together, these elements form a robust defense and gatekeeper system that prevents the entry of harmful substances and allows beneficial nutrients and molecules to pass through.

When the gut barrier is weakened, whether due to dysbiosis, antibiotic usage, or dietary insufficiencies, prebiotics can help restore its integrity. By selectively nourishing beneficial gut bacteria, prebiotics help these organisms thrive, promoting the production of short-chain fatty acids (SCFAs) such as butyrate. These SCFAs directly enforce epithelial health, strengthen tight junctions, and enhance mucus production. Also, direct effects on epithelial and mucus integrity have been reported for prebiotics [2,3,4].

Additionally, regular intake of prebiotics can contribute to the long-term resilience of the gut barrier, making it less susceptible to disruptions from dietary changes or external stressors. Through this ongoing support, prebiotics give rise to a balanced microbial environment and the gut’s essential protective functions, ensuring a healthier, more resilient intestinal system over time [5,6].

Gut barrier disruption may lead to substantial intestinal tissue inflammation due to an unnatural over-interaction between bacterial-derived molecules and the host immune system apparatus. While a typical gut environment has a basal stimulation of immune cells, resulting in a ready-to-act immune system, high inflammation can lead to problems such as metabolic disorders, higher toxicity (due to increased permeability), and colon carcinogenesis [5].

In addition to prebiotics, probiotics might also support health. Probiotics are defined as beneficial microorganisms consumed to preserve and promote gut health. According to Patra et al. (2022), through a robust systematic network and meta-analysis, probiotics or probiotic-derived bacteriocins could interact directly with immune enzymes involved in colorectal cancer (CRC) pathogenesis, such as COX-2, and even modulate nod-like receptor protein-3 (NLRP3) or NF-kB pathways, reducing CRC-associated inflammation [7].

The aim of this review is to elucidate, based on the literature, if those crucial interactions with immune and metabolic receptors also happen with prebiotics. While some prebiotics have been extensively studied in preclinical models, this review focuses on updating the current understanding of both emerging and conventional prebiotics. Specifically, it explores their role in enhancing gut barrier integrity, reducing inflammation, and mitigating the risk of colon cancer. This review aims to connect findings from preclinical research with evidence from clinical trials and human studies. By doing so, it provides a thorough analysis of the effectiveness of prebiotics and their potential applications for enhancing human health.

## 2. Bibliometric Analysis Methodology

For the present analysis, the Scopus database was utilized mainly due to its excellent and intuitive interface for replicating such criteria described here in the future. Two sets of criteria were used. The first focused on tracking the level of interest and the publication rates of reviews and articles on prebiotics over time. The following three sets of keywords were used in this first approach, merged by the Boolean operator “AND”:

(1) “prebiotics” AND “inflammation”;
(2) “prebiotics” AND “colon” AND “cancer”;
(3) “prebiotics” AND “TLR”.

The time of publication was set to start in 1997, the first year reporting any publication for all three search settings. The language was limited to English for its position as the universal academic language. Finally, the subject areas chosen to restrict the search were focused on those more inclined to interfere with health-related matters regarding prebiotics, including “Medicine”; “Biochemistry, Genetics, and Molecular Biology”; “Immunology and Microbiology”; “Pharmacology, Toxicology, and Pharmaceutics”; and “Chemistry”.

**Figure 1:**
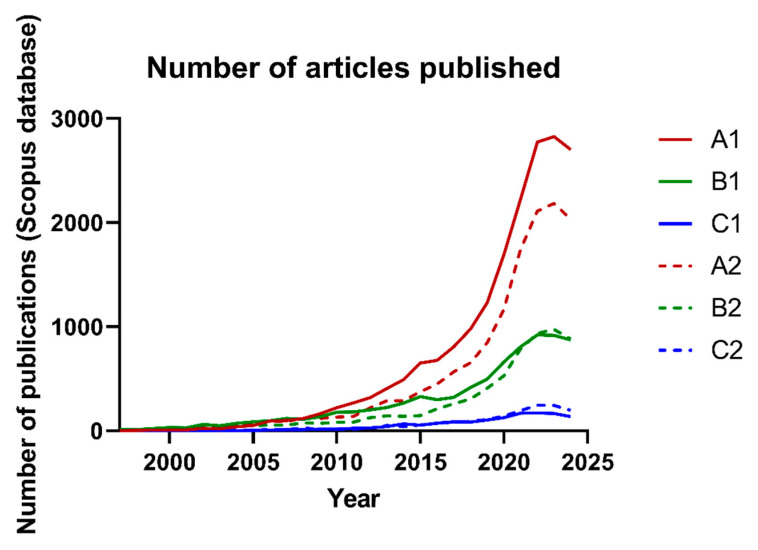
Number of articles published in the Scopus database starting 1997 until now. Number 2 (dotted lines) represents the number of “review articles” under a determined set of terms, while number 1 (solid lines) shows the number of “original articles”. A: “prebiotics” AND “inflammation”; B: “prebiotics” AND “colon” AND “cancer”; C: “prebiotics” AND “TLR”.

The purpose of Figure 1 is to show the comparisons between different search terms and the number of reviews (indicated by letters followed by the number 2 and represented with dotted lines) and original articles (indicated by letters followed by the number 1 and represented with solid lines) for each term. As visualized in Figure 1, “inflammation” is a search term that appears more often than the other search strings in any given year. This also comes from a terminology point of view since inflammation is a significant umbrella term that, aside from being studied by itself, supports several physiological and pathological processes involving—or not—human health issues. Nevertheless, from this, we can expect that such terminology often resonates more in the literature due to its relevance and complexity and as a “primary pillar” topic. Terminology C, “prebiotics and TLR” is a more straightforward, smaller concept within the larger “inflammation topic” and has kept quite a small and constant uprising, especially from 2020 onwards. It is assumed that this topic may have gained attention through its connection with other critical subjects. These include immunotherapy research focused on immune receptors, the interaction of specific prebiotics with these receptors, and a growing interest in tight junction proteins, gut barrier stability, and related pathologies. Additionally, terminology B, “prebiotics and colon cancer”, although potentially associated with these areas, has a distinct biomedical background. This unique background supports methodological independence, encompassing research into chromosomal instabilities, genetic disorders, potential surgical treatments, and other related factors. Even then, specific trends are similar for all the terms used, at least when using the methodology shown here, with particular attention to a plateau of publications observed from 2022 to 2024. This presentation offers an overview tailored to both researchers and lay readers. It highlights the emerging publication trends over recent years, identifies areas with limited or underexplored literature, and pinpoints sharp increases in interest around specific keywords. Additionally, it aids in constructing VOSviewer map inputs and refining subsequent searches. For example, interest in the interaction between TLRs) and prebiotics has shown a gradual but modest increase. This trend lacks the significant surge observed between 2016 and 2021 for the broader keyword “inflammation”. In the last four years, the topic has reached a plateau and has even been surpassed by reviews focusing on the same subject.

The second search approach was similar to the one described above, except for the publication date being set between 2021 and 2025, to select only for very recent literature for the VOSViewer software analysis. The Scopus database search document data were exported as .csv files using all the citation, bibliographical, and abstract/keyword data. Using the VOSViewer software, version 1.6.18, a map was created based on bibliographical data. With the Scopus file extension selected, a co-occurrence of all keywords with complete counting was performed, and the simulations were chosen based on a minimum occurrence of 100 times for all the terms searched. Visualization was standard, with attraction and repulsion values set as the default.

**Figure 2:**
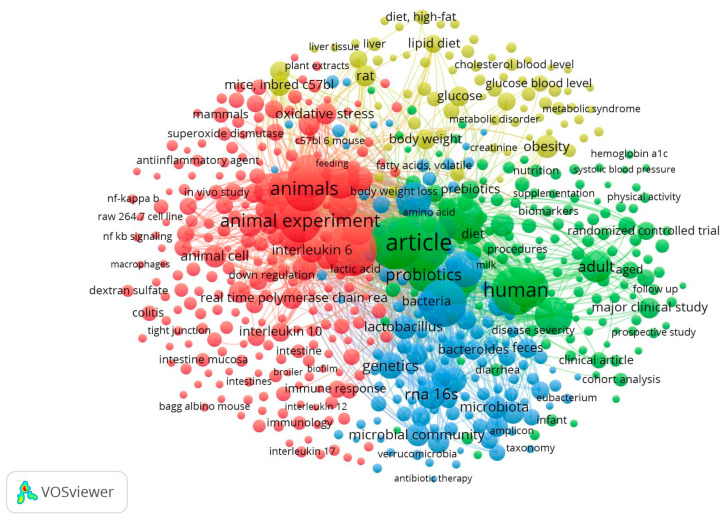
VOSViewer map from the search combination of the terms “prebiotics” and “inflammation” (search A).

**Figure 3:**
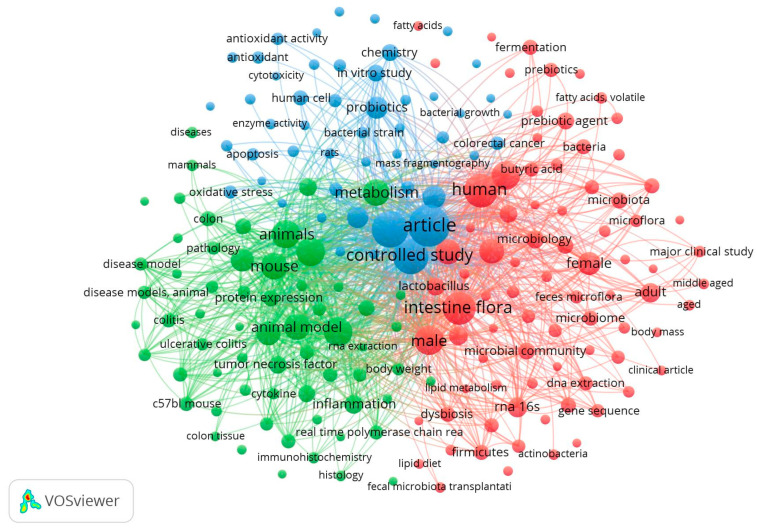
VOSViewer map from the search combination of the terms “prebiotics” and “colon” and “cancer” (search B).

**Figure 4:**
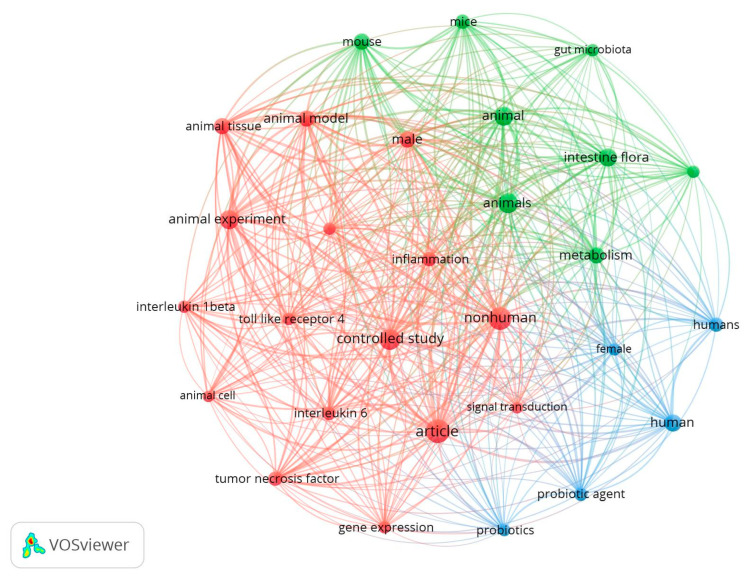
VOSViewer map from the search combination of the terms “prebiotics” and “TLR” (search C).

Such relevance of the data, briefly discussed above, can be observed from the difference in densification among Figure 2, Figure 3 and Figure 4. Figure 2 shows the result from the search “A” (prebiotics and inflammation), which shows an intensely dense formation of four keyword clusters. Each cluster is well-defined, and boundaries can be drawn.

The red cluster is focused on animal and cell experimentation data and methodology, ranging from animal model types, such as “albino mouse”, “inbred c57”, and “raw 264.7 cell line”, which refer not only to animal and cell types but also to how the study is set as an intervention/condition, as shown by terms such as “colitis” and “dextran sulfate”.

The green cluster holds terms related to human clinical studies, with both methodologies such as “cohort analysis”, “randomized clinical trial”, and “prospective study”, as well as population characteristics, such as “adult”, “aged”, “supplementation”, and “diet”.

The blue cluster is mainly focused on microbiome-related terms, which was highly expected to be relevant. Some of the terms include “*Bacteroides*”, “*Verrucomicrobia*”, and “*Lactobacillus*”.

Finally, the yellow cluster is the smallest one and, interestingly, highly related to metabolic syndromes, with terms such as “liver tissue”, “glucose”, “cholesterol”, blood levels, “metabolic disorder”, among others.

Figure 2 shows the proximity between the microbiome-related and metabolic disorder terms, intermediated by inflammation-related keywords. Based on that, additional research with the same parameters as described for the maps but changing the search terms to “prebiotics AND microbiome AND metabolic disorder”, as well as excluding two reoccurring “contamination” terms through the usage of the “AND NOT” Boolean operator (probiotics and review), resulted in 407 original article documents in the last five years in the Scopus database, giving us an average of about 81 original articles exploring such correlations per year. Examples of metabolic disorders identified were glucose tolerance, cardiomyopathies, insulin resistance, and hyperlipidemia.

Regarding search “B” (prebiotics and colon cancer), three less dense clusters reflecting the lower amount of published data available are highlighted in Figure 3.

The Green cluster is related to the animal modeling and analytical methodology used, with terms such as “ulcerative colitis” (a risk factor for colorectal cancer—CRC), “immunohistochemistry”, “histology”, and “RNA extraction”.

The red cluster merges terms related to human clinical studies (“middle-aged”, “major clinical study”, and “female”), the microbiome (“prebiotics”, “lactobacillus”, “intestine flora”, and “dysbiosis”), and finally microbiome-related methodologies (“RNA 16s”, “fecal microbiota transplantation”, and “DNA extraction”.

The blue cluster is mainly related to in vitro methodologies such as “mass fragmentography”, “apoptosis”, “enzyme activity”, and “antioxidant activity”.

We applied the above exclusion criteria to check the literature convergence between microbiome, colorectal cancer, and prebiotics. We obtained 258 original article papers over the past five years (averaging 52 papers/year). Similar to what is observed in Figure 3, the search results were intrinsically connected to inflammation and fermentation. In this search, a common approach to colorectal cancer evaluation in animal models, “aberrant crypt foci”, was also identified.

Figure 4 shows the scarcity of terms with similar levels of relevance/occurrence when using the combination of terms “prebiotics” and “TLR”. Nevertheless, a particular pattern is maintained due to their natural separations between animal experimentation and human studies (blue cluster). But this time, the red and green clusters were, albeit separated, highly related and similar, with terms shared between them such as “animal experiment”, “mice”, “animals”, among others. Complementary terms related to the microbiome, such as “intestine flora”, were under the green cluster. Meanwhile, terms more related to inflammation, such as “toll-like receptor 4”, “interleukin-6”, and “tumor necrosis factor”, were under the red cluster.

Despite the scarcity, one final search was performed to identify the proximity relationships among the microbiome-, TLR-, and prebiotic-related terms. Here, the findings were reduced, with 62 original articles published over the last five years, which was an average of about 12 articles per year. Again, converging with the terms found in Figure 4, many articles explored TNF-α expression/secretion (red cluster). Although not present in the figure, butyrate production was also relevant in the search, alongside high-fat diet-induced obesity, epithelium integrity, and immune function.

The following sections were written based on the articles in the above bibliometric analyses.

## 3. Prebiotics Definition, Types, and Effects

While the first definition of prebiotics can be tracked to as early as three decades ago, more updated versions have been distributed throughout the literature. The most accepted updated definition is the one from ISAPP (International Scientific Association for Probiotics and Prebiotics), which describes prebiotics as “substrates that are selectively utilized by host microorganisms conferring a health benefit” [8]. The World Gastroenterology Organization Global Guidelines specify the following two extra points: (1) selective fermentation, not general “utilization”; and (2) resulting in compositional or activity changes of the gastrointestinal microbiota [9].

**Figure 5:**
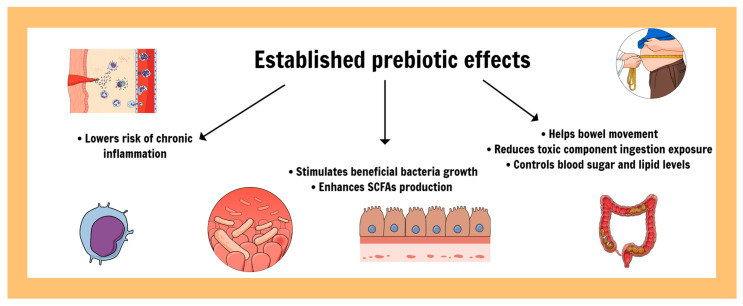
Established prebiotic effects for intestinal and systemic health.

The most usual prebiotics are β-glucans, pectins, fructo- and galactooligosaccharides (FOSs and GOSs), inulins, lactulose, and human milk oligosaccharides (HMOs). Other fermentable polysaccharides can also be characterized as prebiotics [9,10]. Polyphenols, resistant starch, and conjugated linoleic acids are also being studied as potential candidates for being described as prebiotics, further expanding this realm [8,11]. Being non-digestible is a requirement since it must be available for use by the beneficial bacteria in the host intestinal tract. Such molecules naturally occur in healthy diets, either present in whole grain foods, vegetables, and fruits, but they can also be supplemented [10]; some of the classic beneficial effects of this compound class are shown in Figure 5.

The chemical structure of a substance is a key factor in determining whether it qualifies as a prebiotic. More complex structures present challenges because they are harder to standardize, making it difficult to predict their effectiveness when used for prevention or treatment. The term “prebiotics” is not limited to carbohydrate-based molecules. In fact, many dietary fibers can exert prebiotic effects, depending on their fermentability. Simpler sugars, such as fructans and glucans, are already recognized as prebiotics. However, more complex structures, like heteropolysaccharides (for example, pectins), can also qualify as prebiotics. For those, some factors such as high molecular variability can be influenced by the extraction method, source, molecular size, and the availability of glycosidic bonds necessary for bacterial breakdown, changing their prebiotic behavior [12].

### 3.1. Gut Barrier Function

The gut barrier is a complex, multi-layered system crucial in maintaining gut homeostasis and protecting the body from external injuries. It comprises a physical barrier of the gut microbiota, a mucus layer, epithelial cells, and immune cells, working together to prevent bacterial adhesion and regulate other processes [13,14]. The gut microbiota keeps the host healthy by competing with pathogenic and symbiotic organisms. They compete for space and resources, primarily derived from diet, to grow and expand their populations at the expense of pathogens [15]. In the mucus layer, antimicrobial products and secretory IgA are released, and its thickness can be regulated via the proteolytic activity ratio of bacteria and the secretory capability of goblet cells [16].

As one of the mentioned parts of the gut barrier, epithelial cells form an intestinal epithelial barrier (IEB). This IEB serves as a critical interface between the external environment and the body’s internal milieu, regulating the passage of nutrients and preventing the entry of harmful antigens and microorganisms [17]. Tight junctions (TJs) within the IEB are essential to controlling paracellular permeability and maintaining barrier integrity. When TJs are dysfunctional, they lead to increased gut permeability, generating the so-called “leaky gut syndrome” [17,18].

Inflammation is closely connected to the function and stability of the gut barrier. For instance, an imbalance in the gut microbiota can disrupt the epithelial barrier by breaking down the mucus layer or causing cellular stress through exposure to the underlying epithelial cell layer. Conversely, certain beneficial strains can produce SCFAs from fermentable substances, and they provide a rapid energy source for epithelial cells, which can help strengthen both the cellular and mucus layers of the gut barrier [19].

The local immune system is also influenced by antigens derived from the microbiota. Regulatory T cells (Tregs) with a tissue repair phenotype, known as ST2+, make up a significant portion of the Foxp3+ Treg population in the gut’s lamina propria. ST2 interacts and responds to IL-33, an alarmin, and amphiregulin (AREG), a growth factor. Overall, Tregs help reduce the intensity of proinflammatory responses to common microbial molecules. For example, Tregs use aryl hydrocarbon receptors (AhRs) to increase basal resistance and minimize tissue damage, promoting a more controlled immune response [20,21].

### 3.2. Inflammation and Carcinogenesis

Colon cancer is one of the most prevalent and lethal types of human cancer, according to the Globocan statistics [22]. The interplays between environmental interferences and genetic alterations heavily induce its pathogenesis. For instance, chromosomal instability (CIN) is a hallmark of most colon tumors and significantly contributes to tumor progression by increasing genetic abnormalities. Errors during mitosis and issues with spindle checkpoint activity often facilitate CIN, resulting in aneuploidy and the loss of heterozygosity. CIN can be triggered by chromosomal segregation molecules, such as aurora kinase B (AURKB), which is activated by BOP1. BOP1 is stabilized by the overexpression of the long noncoding RNA CCAT2. Additionally, epigenetic factors may influence this process. Certain microRNAs (miRNAs) can regulate epithelial–mesenchymal transition, a mechanism linked to the onset of colorectal cancer (CRC) [23].

The tumor microenvironment comprises immune cells, stromal cells, and the intestinal microbiome, all playing a role in CRC pathogenesis. For example, MAPK-activated protein kinase 2 (MK2) in macrophages has been linked to a pivotal contribution to colon tumorigenesis under chronic inflammation. Tumor angiogenesis (vascularization) is one of the consequences of the MK2 activity [24,25]. Chronic inflammation is a significant risk factor for the development of colorectal cancer (CRC). Patients with chronic inflammatory bowel diseases, such as ulcerative colitis and Crohn’s disease, face an elevated risk of developing colitis-associated cancer (CAC). The persistent inflammatory state in the colon is strongly associated with tumor development, and the risk of CAC increases with the duration of inflammation [26,27].

Some critical immune signaling pathways are implicated in the pathogenesis of inflammation-associated colon cancer. The NF-kB pathway, for example, is crucial for regulating immune responses and inflammation. Still, its activation promotes gene expression in cell proliferation and survival, which can contribute to tumorigenesis. Prostaglandins and cyclooxygenases have also been linked to the development of CRC. Moreover, cell survival and strength can be promoted by the STAT3 signaling pathway, which can be activated via IL-6, a proinflammatory cytokine [26].

Cytokines are essential mediators of the immune response and can influence colorectal cancer (CRC) development. TNF-α and IL-1β are key inflammatory cytokines that promote CRC progression by stimulating interactions between various cell types in the gut. Maintaining a balance between pro- and anti-inflammatory cytokines is crucial for preserving tissue homeostasis and preventing malignant transformations [28,29].

As briefly mentioned before, the gut microbiota significantly influences the inflammatory environment in the colon, and alterations in the diversity and function of the microbiome are associated with changes in the immune response. The toll-like receptors (TLRs) on immune cells respond to microbial components, promoting tolerance or activating inflammatory signaling pathways, leading to CRC. Consequently, the microbiota and its metabolites are part of a “microbiome-inflammation” axis [27,30,31].

### 3.3. Prebiotics and Inflammation: A Clinical View

Targeting inflammatory pathways and cytokines is a therapeutic strategy to prevent or treat inflammation-associated CRC. The use of monoclonal antibodies, tyrosine kinase inhibitors (e.g., cetuximab), and nucleic acid drugs (e.g., siRNAs and antisense oligonucleotides) as modulators of inflammatory responses is already a trend in clinical exploration for not only CRC but other types of cancers as well [32,33,34,35,36].

However, in this review, we propose to evaluate whether prebiotics can significantly impact inflammation and CRC as well. Postbiotics, which are a combination of probiotics and prebiotics, such as butyrate (an SCFA), can modulate specific immune cells. For example, Kang et al. (2023) found out through a big-cohort study that butyrate boosted anti- programmed cell death protein 1 (PD-1) efficacy by inducing functional CD8+ T cells. PD-1 is considered an immune system “brake” protein, limiting immune response throughout CRC development [37].

Polyphenols are also classified as potential prebiotic candidates. Molinari and colleagues (2021) pointed out that human clinical trials analyzing such treatments are rather scarce, primarily focusing on inflammatory biomarkers, as opposed to animal studies, which the authors deeply dive into [38]. Nevertheless, Macis et al. (2023) tested both curcumin and anthocyanin commercial formulations in a phase II presurgical trial in patients with adenomatous polyps. Still, they did not achieve statistical significance related to the inflammatory biomarkers evaluated (IL-6, IL-10, and TNF- α) [39]. Moorthy et al. (2021) reported in their systematic review, specifically taking animal studies into account, that almost all studies sampled with polyphenolic extracts and all those utilizing pure polyphenols resulted in improvements in the metabolic and gut microbiota profiles. Gut microbiota genus level abundances were correlated to the symptom-alleviating trend after high-fat diet-induced obesity, while alpha diversity was not altered consistently throughout the gathered literature [40].

Another type of emerging prebiotic is resistant starch (RS), which is often a product of the starch retrogradation process but can also be found naturally in some grains, beans, and green fruits. In a placebo-controlled clinical trial, a patented resistant potato starch (Solnul^TM^) reduced diarrhea and constipation effects compared to the control and significantly increased *Akkermansia* and *Bifidobacterium* levels [41]. Within in vitro digestion and fermentation simulations, mixed bacterial cultures exhibited significant growth compared to the control. Notably, the growth of *Lactobacillus* and *Bifidobacterium* mono-cultures was particularly enhanced by RS [42]. Technologies to extract more RS from different sources continue to be explored. Das et al. (2022) reported an enzyme-mediated biotransformation that doubled the RS yield. The RS-rich banana flour generated had a better prebiotic and symbiotic potential, enhancing the growth of the *Bacillus coagulans*, *Lactobacillus rhamnosus*, and *Saccharomyces boulardii* strains [43]. Notably, those prebiotics can often be employed as tools for other purposes, such as carrying compounds that are sensitive to the upper gastric chemical environment, as happens with RS nanoparticles. Wang et al. (2022) found improvements in *Lactobacillus plantarum* growth, its S-layer protein stability, as well as higher butyric acid levels after the addition of RS nanoparticles in a 0.5% concentration [44].

Conventional prebiotics may benefit inflammatory-derived health conditions, such as psoriasis, which are not typically associated with gut health. Buhas et al. (2023) found that after 12 weeks of consuming probiotics and prebiotics, an intervention group of 42 subjects showed significant improvements in the quality-of-life scores related to dermatological issues. Regarding inflammatory markers, TNF-α significantly decreased in the intervention group, while IL-10, an anti-inflammatory cytokine, significantly increased. Although IL-6 and IL-17a showed numerical changes, these were not statistically significant [45].

The symbiotic administration of *Bifidobacterium animalis* and fructo-oligosaccharides (FOS) for 30 days in middle-aged subjects impacted systemic inflammation, where IL-6, IL-8, IL-17a, and IFNγ were significantly reduced in the plasma samples. Such effects were not observed in the placebo group, and the authors stated that it was probably gut permeability-independent results due to the lack of change in fecal albumin or plasma intestinal fatty acid-binding protein (iFABP) presence [46].

In an elderly population, the symbiotic effect of *Bifidubacterium animalis* subsp. *lactis* and inulin that focused on cognitive function improvements also achieved the relevant changes regarding inflammation biomarkers. A significant increase in the leukemia inhibitory factor receptor (LIR), CCL-23, and TNF ligand superfamily member 12, as well as a decrease in sulfotransferase 1A1, was detected [47]. CCL-23, for example, is a chemotactic cytokine for resting T cells, monocytes, and neutrophils. This could signify a proinflammatory treatment profile, contrary to what is often expected.

De Giani et al. (2022) compared the effects of administering prebiotics alone versus a symbiotic treatment that included two strains of *Lactobacillus*, one strain of *Bifidobacterium*, and two types of fructans (DP 3-5 and DP10 inulin). In elderly participants, they observed that those in the symbiotic group showed increased levels of fecal calprotectin and β-defensin 2 after 28 days (T28), which remained elevated at a 28-day follow-up (T56). In contrast, the prebiotic group showed an increase in β-defensin 2 at T28, but this effect did not persist to T56. Fecal calprotectin levels in the prebiotic group remained unchanged [48]. Moludi et al. (2021) also reported more significant benefits in groups receiving a combination of probiotics and inulin (symbiotic) than inulin alone or a placebo. While the inulin-only group showed a modest increase in the anti-inflammatory cytokine IL-10, reduced levels of lipopolysaccharide (LPS; indicating reduced microbial translocation), and decreased TNF-α, the symbiotic treatment produced much more potent effects. Specifically, the symbiotic group had increases of 0.37 and 10.10 ng/mL for IL-10, reductions of −1.86 and −22.02 EU/L for LPS, and reductions of −5.73 and −25.05 ng/mL for TNF-α in the inulin-only and symbiotic groups, respectively [49].

In a study by Neyrinck et al. (2021) involving obese patients, fecal calprotectin levels were reduced in the intervention group despite using only inulin, without any probiotics. This finding suggests that inulin alone may help reduce gut inflammation. However, the treatment did not alter the composition of SCFAs. Interestingly, rumenic acid, a conjugated linoleic acid with immunomodulatory potential, was increased in the fecal content and was correlated with an increased abundance of *Bifidobacterium* [46]. In another study involving obese children, inulin administration for up to six months did not impact fecal calprotectin levels or cytokines like IL-1β and IL-6 [50]. The variability in outcomes across these studies may have resulted from significant population differences, highlighting the need for further comparable research to confirm these effects.

A meta-analysis of randomized controlled trials investigated the effects of prebiotics, probiotics, and symbiotics on patients with non-alcoholic fatty liver disease (NAFLD). Pan et al. (2024) found that prebiotics alone can reduce inflammation by lowering TNF- α, IL-6, and C-reactive protein (CRP) levels [51]. Apart from being related to metabolic and liver health, they can also increase local inflammation in the large intestine, compromising gut health.

### 3.4. Prebiotics and TLRs: Direct Modulation Matters?

**Figure 6:**
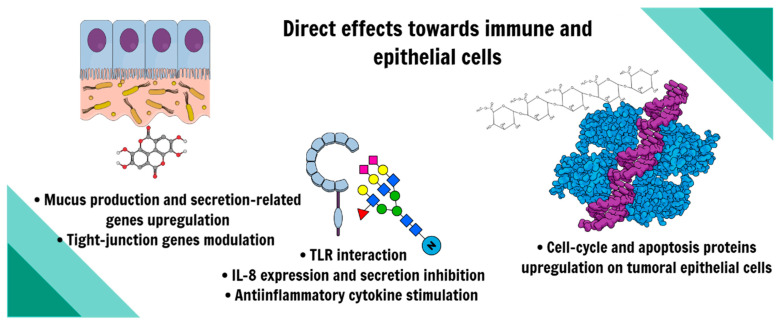
Direct effects observed of prebiotics in both immune and epithelial cells.

Several preclinical studies have elucidated another aspect of dietary prebiotics, especially the ones of carbohydrate origin, which is the role of the direct modulation of immune receptors and other molecules. Toll-like receptors (TLRs), for example, are vital triggers for innate immune responses and are necessary for microbial tolerance in the intestine, but they have also been implicated in other biological scenarios. What if the inflammatory marker outcomes observed and discussed in the previous topic could also have a role in this direct modulation and are not only driven by microbiota regulation? A summary of the observed outcomes is shown in Figure 6.

A robust cross-sectional study evaluating over 14,000 participants found (after adjustments towards age, gender, race, and other biological parameters) inverse associations of dietary fiber (including some prebiotics) intake and systemic inflammation index, systemic inflammation response index, neutrophil-to-lymphocyte ratio, and other immune-related metrics [52]. The authors argued for both sides; although the microbiota changes caused by prebiotics are established mechanisms, the significant contribution of the direct effects from dietary fiber in the immune system is a particularly interesting trending topic.

Imiquimod is a pharmacological agonist of TLR7, and there is a psoriasis-like induction model of rats with the topical application of imiquimod. A diet containing inulin administered to the animals improved skin thickness, erythema, scaling, and other visual aspects of inflammation while decreasing the number of inflammatory-infiltrating cells. The authors, however, focused on microbiota modulation and concluded that propionate production, at least partly, plays a role in attenuating inflammation. However, the inhibition of epidermal hyperproliferation was only achieved through the high-inulin diet and not with oral propionate ingestion, influenced by keratin-16 expression [53]. The mechanisms, nevertheless, still need to be more conclusive.

By using inulin as a treatment approach, the integrity of the blood–brain barrier (BBB) and inflammation after chronic stress were restored and managed by the inclusion of this prebiotic. TJ protein expression in the gut barrier, such as claudin-5, VE-cadherin, occludin, claudin-1, and ZO-1, were higher in the inulin group than in the condition-positive group. Aside from the structural help, TLR4 abundance and NF-kB were higher in the condition-positive group, leading to a higher release of iNOS, COX-2, and TNF- α. A reversion of this scenario was observed using inulin treatment. Once more, inulin enhanced the SCFA levels in the feces and serum, corroborating data from other studies. Therefore, modulation of the TLR4/MyD88/NF-kB pathway was pointed out as the most influenced by inulin treatment in chronically stressed mice [54].

Metabolic syndrome-related inflammation (on systemic adipose tissue) was also controlled in rats that ingested 10% prebiotic inulin. Adipocyte area decreased, alongside lower mRNA expression of IL-6 and TLR4 transmembrane protein, which led to a better inflammatory profile of the epididymal adipose tissue of the animals [55]. Different fructans, such as inulin-like, levans, and graminans, also performed as immune modulators in several in vitro experiments. Akkerman et al. (2024) found that fructose-based levan exopolysaccharides had a molecular-weight-dependent activation on TLR2 and 4 dependently on the MyD88 adapter molecule. Inhibition of TLR5 and 8 was also achieved, showing both pro- and anti-inflammatory potentials [56]. Meanwhile, in another study, graminan-type fructans (GTFs) activated TLR3, 7, and 9, and inhibited TLR2 and 4. Coincidently, inulin-like fructans (ITFs) activated TLR2 and 4 but did not exert inhibition of any particular TLR. While stimulating dendritic cells, GTFs and long-chain ITFs reduced proinflammatory cytokine production, such as TNF-α and MCP-1 [57]. Activation needs to be considered since some types of patients, for example, those with inflammatory bowel disease, may not be able to tolerate fructan fibers that are not totally fermented, potentially leading to impairment of the condition [58].

The most commonly studied prebiotics are inulin, FOS, and GOS. Their relatively simple structures facilitate regulatory approval and standardization for population health. When considering other types of dietary fibers, the structural differences between molecules play a significant role in interactions with TLRs. For example, Lagos et al. (2024) found that type B-resistant starch crystals led to higher TLR2-dependent NF-κB activation in both in vitro and in silico experiments. TLR4 activation was also observed exclusively with these B-type crystal structures, while no activation was detected for TLR5. In monocytes, this activation triggered the release of IL-8, TNF-α, and IL-1RA. Structural factors such as polydispersity, crystallinity, and chain length are components that may be adjusted to achieve targeted activation of specific receptors [59].

Pectins are also candidates for direct immunomodulation, especially regarding TLR interaction. One of the main structural components for pectins in this regard has been the degree of methylation, which is the proportion of methylated galacturonic acid (Gal*p*A) units throughout the backbone, and the degree of blockiness, which is the interval ratio of methylations in the pectic chain. TLR2/1 dimerization was better inhibited through blocks of non-methylated Gal*p*A units intercalated with methylations [60]. Gasaly et al. (2024) identified that both low and high DM lemon pectins inhibited the IL-8 secretion induced by TLR2/1, with low DM pectin being more efficient. Also, low DM pectin was still the most potent anti-inflammatory and suitable for TJ protein gene expression modulation [61]. The inhibition was more pronounced for both samples in the Caco-2 cell line and less in the DLD-1 cell line. While both lemon pectin samples differed between high and low DM, both had a blockwise distribution of those methyl groups, which can form anchor points when binding to some TLR pockets [61]. Pam3csk4 (TLR2/1 agonist) enhanced claudin-1 and 3 in both cell lines while enhancing claudin-2 only in the DLD-1 cell line. Those trends were reversed by low DM pectin, depending on the cell type, but not by high DM pectin [61].

Li et al. (2023) administered chows enriched with long-chain 57% DM citrus pectin for mice with *S. typhimurium*-induced colitis. The animals exhibited a reversal of colon shortening and improved disease activity index (DAI) scores. TJ proteins, including occludin, ZO-1, and ZO-2, downregulated by colitis, had their expression restored after pectin treatment. Additionally, the upregulation of TLR2 and NF-κB was reversed in the pectin-treated group [62]. It is important to note that all the studies mentioned above used pectins with high levels of Gal*p*A residues rather than neutral sugars like those found in rhamnogalacturonan (RG) fragments. Hyun et al. (2023) conducted an in vitro and in silico analysis of pectin heteropolysaccharides and discovered that pectin galactans are strongly affixed to the leucine-rich segment of TLR4. Interestingly, these fragments selectively bind to TLR4 and not TLR2, which activates macrophages toward an inflammatory profile [63]. Structure–function relationship studies of these carbohydrates are essential to understanding these variations in the effects of different pectic fragments. This approach will enable more refined preclinical and clinical testing in the near future.

A lesser-known prebiotic fiber, konjac glucomannan (KGM), was also shown to directly impact the immune system of mice beyond microbiota regulation. Once again, TJ proteins, such as occluding and ZO-2, in the gut were upregulated after KGM treatment, and the overall colonic barrier was enhanced following the pretreatment of KGM and injection of *S. typhimurium* (to induce colitis). The proposed mechanism was via TLR2, not via TLR4, resulting in the improved upregulation of TNF- α and CCL8, as well as the downregulation of IFNβ. Of course, some of the effects observed were also correlated but not limited to microbiota regulation [64].

## 4. Conclusions and Future Research

Overall, prebiotic consumption has been widely studied as a potential strategy for reducing inflammation by modulating the intestinal microbiota, with research often focusing on the development and progression of colorectal cancer (CRC) through in vitro and in vivo studies, as well as clinical trials. Although the definition of prebiotics varies across the literature [8,9], it generally includes dietary fibers, fructo- and galactooligosaccharides, specific conjugated lipids, and emerging candidates like polyphenols [38,46,48]. Clinical trials in humans have shown that various prebiotic and symbiotic treatments frequently modulate inflammatory markers such as IL-6, IL-10, and TNF-α. Such use aims to improve gut barrier function and structure, immune tolerance, and reasonable immune response buildup and is a promising alternative for co-adjuvant therapy. New studies have shown that some prebiotics can directly interact with cells from the immune system and the gut epithelial barrier, making the biological effects of prebiotics go beyond microbiota modulation. Nevertheless, some interindividual differences are limitations of a significant part of human clinical studies; therefore, more work focusing on avoiding and lowering such limitations is necessary for the progression of prebiotic usage protocol establishment. Hopefully, in the near future, it will be possible to further explore how particular prebiotics directly modulate immune molecules, enhancing our understanding of the impact this may have on specific immune-related disorders.

Future research on prebiotics should aim to provide more definitive evidence of both microbiota-dependent and direct immune-modulating effects, as these interactions offer promising therapeutic potential. The ability of prebiotics to support gut health and reduce inflammation has been well documented, primarily through microbiota modulation. However, emerging studies have revealed that prebiotics may also interact directly with immune cells and the epithelial barrier, suggesting a more complex biological role beyond microbiota influence alone. To build a stronger foundation for clinical applications, future studies should focus on understanding the structure–function relationship of diverse prebiotics and identifying which molecular features are the most effective for specific immune pathways. Standardizing methodologies and considering individual variability will also be crucial, as these factors often introduce limitations in clinical trials. By addressing these areas, future research can establish more explicit protocols for using prebiotics to target immune-related disorders, moving towards their potential role as adjunct therapies in managing inflammation and the associated diseases. Also worth mentioning are the applications of emerging prebiotics such as polyphenols and resistant starch, as well as the technologies such as the nanoparticle carriage system, especially in human studies, which should also contribute to further advances.

Overall, prebiotics are promising for the enhancement of health and resilience, especially by supporting gut integrity and immune function. This paper highlights evidence that prebiotics can assist in restoring the gut barrier and reducing inflammation, particularly following disturbances such as antibiotic use or other gut injuries. Prebiotics provide a valuable approach to maintaining health and preventing disease in vulnerable individuals by replenishing beneficial microbes and directly strengthening epithelial and immune cell functions.

## Data Availability

Data will be made available upon request.
